# Functional analysis of the stable phosphoproteome reveals cancer vulnerabilities

**DOI:** 10.1093/bioinformatics/btac015

**Published:** 2022-01-07

**Authors:** Di Xiao, Hani Jieun Kim, Ignatius Pang, Pengyi Yang

**Affiliations:** Computational Systems Biology Group, Children’s Medical Research Institute, Faculty of Medicine and Health, The University of Sydney, Sydney 2145, Australia; Computational Systems Biology Group, Children’s Medical Research Institute, Faculty of Medicine and Health, The University of Sydney, Sydney 2145, Australia; Charles Perkins Centre, School of Mathematics and Statistics, The University of Sydney, Sydney 2006, Australia; Bioinformatics Group, Children’s Medical Research Institute, Faculty of Medicine and Health, The University of Sydney, Sydney 2145, Australia; Computational Systems Biology Group, Children’s Medical Research Institute, Faculty of Medicine and Health, The University of Sydney, Sydney 2145, Australia; Charles Perkins Centre, School of Mathematics and Statistics, The University of Sydney, Sydney 2006, Australia

## Abstract

**Motivation:**

The advance of mass spectrometry-based technologies enabled the profiling of the phosphoproteomes of a multitude of cell and tissue types. However, current research primarily focused on investigating the phosphorylation dynamics in specific cell types and experimental conditions, whereas the phosphorylation events that are common across cell/tissue types and stable regardless of experimental conditions are, so far, mostly ignored.

**Results:**

Here, we developed a statistical framework to identify the stable phosphoproteome across 53 human phosphoproteomics datasets, covering 40 cell/tissue types and 194 conditions/treatments. We demonstrate that the stably phosphorylated sites (SPSs) identified from our statistical framework are evolutionarily conserved, functionally important and enriched in a range of core signaling and gene pathways. Particularly, we show that SPSs are highly enriched in the RNA splicing pathway, an essential cellular process in mammalian cells, and frequently disrupted by cancer mutations, suggesting a link between the dysregulation of RNA splicing and cancer development through mutations on SPSs.

**Availability and implementation:**

The source code for data analysis in this study is available from Github repository https://github.com/PYangLab/SPSs under the open-source license of GPL-3. The data used in this study are publicly available (see Section 2.8).

**Supplementary information:**

Supplementary data are available at *Bioinformatics* online.

## 1 Introduction

Protein phosphorylation regulates diverse protein function, including the catalytic activity and stability of proteins, dictates their subcellular localization and controls the dynamics of protein–protein interaction (PPI) (Ubersax and Ferrell, 2007). Working as a molecular switch, this reversible event is one of the most common post-translational modifications (PTMs) and is intricately controlled by the balanced action between kinases and phosphatases (Hunter, 1995). Phosphorylation has a broad impact on cellular processes spanning from cell cycle progression, alternative splicing, cell differentiation and apoptosis (Ubersax and Ferrell, 2007). The dysfunction of phosphorylation can therefore severely disrupt cellular homeostasis, leading to various diseases (Su *et al.*, 2019) and cancer (Rush *et al.*, 2005). Whilst most research been carried out so far primarily focused on investigating the cell-type- and condition-specific regulation of phosphorylation sites and their functional significance in various disease and physiological states, the stable phosphorylation events that cut across cell types, tissues, conditions and perturbations and the significance of the stable phosphoproteome in cell physiology remains largely unexplored.

Recent advances in mass spectrometry-based technologies (Choudhary and Mann, 2010) and the growing accumulation of phosphorylation-specific resources (Gnad *et al.*, 2007; Hornbeck *et al.*, 2015; Krassowski *et al.*, 2021) and databases (Bodenmiller *et al.*, 2008; Yu *et al.*, 2019) offer a great opportunity to investigate the stable phosphoproteome. Previously, we identified a set of stably phosphorylated sites (SPSs) by using four mouse phosphoproteomics datasets and demonstrated the utility of the stable sites for data normalization and integration (Kim *et al.*, 2021). Whilst our study highlighted the usefulness of these stable sites for phosphoproteomics data analysis, it lacked any systematic and functional characterization of the SPSs themselves.

Here, we hypothesize that, akin to stably expressed genes such as housekeeping genes (Lin *et al.*, 2019), phosphosites that remain stably phosphorylated irrespective of cellular origins and states may represent the core set of phosphoproteome that are tightly regulated to support essential cellular function and homeostasis in a wide range of cell and tissue types, and the dysregulation of such phosphosites could lead to various diseases such as cancer. To systematically identify and characterize the stable phosphoproteome, we developed an analytical framework to identify SPSs from a comprehensive collection of 53 high-quality human phosphoproteomics datasets encompassing a total of 40 cell types/tissues and 194 conditions/perturbations. We validated the stability of our high-confidence human SPSs on three independent datasets, highlighting the cross-species conservation of the SPSs between mouse and human. Further characterization of SPSs based on a diverse set of features ranging from phosphosite- to gene/protein-level information revealed the functional importance and evolutionary conservation of SPSs. We next demonstrated through enrichment analyses a strong association between SPSs and their host proteins with RNA splicing. Consistent with our enrichment analyses, a closer examination of the phosphosites unveiled known and putative phosphorylation events involved in spliceosome assembly and function. Strikingly, we observed that the majority of the spliceosome-associated SPSs are affected by cancer mutations, suggesting their potential impact on spliceosome formation and function. Collectively, our statistical framework provides an effective approach for identifying stable phosphoproteome and the subsequent analyses of SPSs derived from this framework reveal their functional importance across cell types and species and highlighting a potential link between the malfunction of spliceosome and cancer development via mutations on spliceosome-associated SPSs.

## 2 Materials and methods

### 2.1 A statistical framework for SPS identification

Motivated by the assumption that SPSs are commonly identified in all cell/tissue types and biological systems (i.e. recurrence), and undergoing minimal changes of phosphorylation level across different biological processes or under a wide range of perturbations (i.e. phosphorylation changes), we developed a statistical framework to identify SPSs that are characterized by these two stability features from 53 high coverage human phosphoproteomic datasets ≥5000 phosphosites and ≥2 conditions relative to control) curated from the qPhos database (http://qphos.cancerbio.info) (Yu *et al.*, 2019) and its updated version ‘qPTM’ (http://qptm.omicsbio.info) (Supplementary Table S1). Specifically, the recurrence of a given phosphosite is simply the number of times it was identified across all datasets and hence ranges from 1 to 53. To quantify the change of phosphorylation for a given phosphosite across all datasets, we first filtered phosphosites keeping those that had been identified in >20% of 53 datasets. Next, we quantified the maximum value of the absolute log2 fold-change across all conditions and treatments/perturbations in each dataset for the sites that passed filtering, quantile normalized these quantifications across all 53 datasets, and finally took the average across all datasets. 

To obtain a statistical significance for the phosphosites with respect to their stability, for each phosphosite, we first fitted a gamma distribution to the values of each of the two stability features, denoted as *X*_1_ and *X*_2_ for recurrence and phosphorylation change, and derived the *P*-values from the upper-tail of the model fitted to *X*_1_ (i.e. recurrence) and lower-tail of the model fitted to *X*_2_ (i.e. phosphorylation change), respectively:

$$P(X_{1}>x)=\int \frac{\beta^{\alpha }}{\Gamma (\alpha )}x^{\alpha -1}e^{-\beta x}dx,$$

and

$$P(X_{2}\leq x)=1-\int \frac{\beta^{\alpha }}{\Gamma (\alpha )}x^{\alpha -1}e^{-\beta x}dx.$$

We then combined the two *P*-values for each phosphosite using Fisher’s method to derive a single statistical significance:

$$P(\chi_{4}^{2}>-2\sum \ln(P))$$

where **P** is a vector of the two *P*-values obtained from the two stability features for each phosphosite.

Phosphosites that have combined *P* < 0.01 were defined as SPSs (Supplementary Table S2). To assess the reproducibility of the proposed analytic framework, first we randomly subsampled (80%) from the 53 datasets, 10 times, and ran the framework to obtain phosphosite stability statistics for each subsample. We assessed the reproducibility by quantifying the concordance of stability statistics between each of all pairs of subsamples using Pearson correlation coefficients. In addition, we also used independent datasets for assessing reproducibility of the framework. These include 22 datasets from qPhos and qPTM databases that have ≥3000 phosphosites and ≥2 conditions (relative to control) (Supplementary Table S1), and are not part of the 53 datasets and four additional datasets that profiles human embryonic stem cells (ESCs), human colon cancer cells (HCT 116), T-cells and human gastric adenocarcinoma cells (AGS).

### 2.2 Evaluating the stability of SPSs

To evaluate the stability of SPSs, we obtained three independent phosphoproteomic datasets, which are not included in the 53 datasets used for SPS identification. These include a human glioblastoma profiling dataset (Recasens *et al.*, 2021) that measures the responses of treatments to glioblastoma cells, and two mouse datasets that profiles mouse embryonic stem cell (ESC) differentiation (Yang *et al.*, 2019) and response of adipocytes to redox signaling (Su *et al.*, 2019), respectively. First, we compared SPSs with size-matched mid- and bottom-ranked sites (*n* = 326), and sites that are not defined as SPSs (i.e. non-SPS) in terms of their maximum absolute log2 fold change in each of the three datasets. Then, for each of the three datasets, we performed principal component analysis (PCA) and hierarchical clustering using either data subset by SPSs or all sites. We quantified the concordance of clustering output with pre-defined labels (time points or conditions) in each dataset using five metrics, including adjusted Rand index (ARI), Fowlkes–Mallows index (FMI), normalized mutual information (NMI), purity and Jaccard index, with the expectation that data subset by SPSs will have significantly lower concordance given they are stably (unchanged) phosphorylated irrespective to time points or conditions (Lin *et al.*, 2019).

### 2.3 Characterization of SPSs

For each of all phosphosites, we derived a diverse set of features from multiple sources to characterize their potential functions, conservations and several other properties on both the phosphosite-level and the gene/protein-level. In particular, the functional scores, the similarity between site flanking region and known kinases position weight matrices, conserved phosphorylation hotspot, the age of inferred ancestral species containing the site, max Netphorest match for all models, and the secondary structure prediction were derived from Ochoa *et al.* (2020). The human or mouse stably expressed gene indexes and gene conservation score for each host gene were obtained from Lin *et al.* (2019). The average protein abundances for each host protein were collected from PaxDb (Wang *et al.*, 2015). For comparison, we included a representative random set, which contains randomly selected non-SPS sites that match the size of SPSs, and all phosphosites in these analyses.

### 2.4 PPI and cancer mutation analyses

To investigate the involvement of SPSs host proteins in PPI networks, we derived high-confidence PPIs from the STRING database (combined score > 900) (Szklarczyk *et al.*, 2019) and the prePPI database (probability > 0.5) (Zhang *et al.*, 2013), respectively. The number of PPIs for each host protein of either SPSs or all phosphosites was quantified and overall distribution compared.

For cancer mutation analysis, we extracted all cancer-associated mutations from the ActiveDriverDB database (Krassowski *et al.*, 2018). This database collates PTMs that have strong links to cancer on the basis of their association with factors such as cancer driver genes using information extracted from the Cancer Genome Atlas (TCGA) (Cancer Genome Atlas Research Network *et al.*, 2013) and the Pan-Cancer Analysis of Whole Genomes (PCAWG) (ICGC/TCGA Pan-Cancer Analysis of Whole Genomes Consortium, 2020) databases. Among the cancer-associated PTMs, we filtered for mutations on phosphosites and calculated the percentage of cancer mutation-affected sites among SPSs and all phosphosites. In addition, we further categorized the mutation frequency of SPS (≥20) and 10 size-matched random sets by cancer types. The mutation counts were extracted from ActiveDriverDB database, which utilized the mutation data of different cancer cohorts from TCGA.

### 2.5 Spliceosome SPS annotation

We annotated a SPS as associated with spliceosome if its host protein is a spliceosomal protein or a splicing-associated protein in either the Reactome (Fabregat *et al.*, 2018) or the KEGG (Kanehisa *et al.*, 2017) databases. For the spliceosome-associated SPSs, we first categorized their host proteins to functional units according to Will and Lührmann (2011), where spliceosomal proteins are annotated for their best known functions in splicing. Next, we categorized the host proteins to eight spliceosomal complexes according to the Spliceosome database (Cvitkovic and Jurica, 2013). We further annotated the spliceosome SPSs that are known to be phosphorylated by CDKs and are affected by cancer mutations in ActiveDriverDB (Krassowski *et al.*, 2018).

### 2.6 Enrichment analyses of pathways, kinases and phosphatase

Enrichment analyses of pathways were performed using Fisher’s exact test for the host genes of SPSs and size-matched random sites against Gene ontology (GO) (The Gene Ontology Consortium, 2017), Reactome (Fabregat *et al.*, 2018), KEGG (Kanehisa *et al.*, 2017) and Biocarta (https://cgap.nci.nih.gov/Pathways/BioCarta_Pathways) databases, and also against cancer gene neighborhoods (CGN) collected from MSigDB (Liberzon *et al.*, 2015). Similarly to pathway enrichment analysis, enrichment of kinases was performed on the phosphosite-level using PhosphoSitePlus (Hornbeck *et al.*, 2015), and phosphatase enrichment analysis was performed on the protein-level using the data derived from Chen *et al.* (2017).

### 2.7 Odds ratio test for cancer mutations

The relative enrichment of cancer mutations in spliceosome-associated SPSs was performed using odds ratio test. The significance of odds ratios and confidence intervals were estimated based on approximation, followed by null-hypothesis (odds ratio equals to 1), as implemented in the fmsb R package (Nakazawa, 2018).

### 2.8 Data availability

The phosphoproteomic data described in this study are publicly available. In particular, the mouse ESC dataset (Yang *et al.*, 2019) (PRIDE: PXD010621), the human glioblastoma dataset (Recasens *et al.*, 2021) (PRIDE: PXD020441) and the mouse adipocyte dataset (Su *et al.*, 2019) (PRIDE: PXD011525) are used for evaluating the stability of selected SPSs. The human ESC dataset (Billing *et al.*, 2019) (PRIDE: PXD004652), the HCT116 dataset (Hahn *et al.*, 2021) (PRIDE: PXD023703), the T-cell dataset (Martinez-Fabregas *et al.*, 2020) (PRIDE: PXD020964) and the AGS cell dataset (Yin *et al.*, 2020) (PRIDE: PXD005093) are included for independent validation of the reproducibility of the proposed framework. All other human phosphoproteomic datasets were curated from the qPhos database (Yu *et al.*, 2019, http://qphos.cancerbio.info) and its updated version (http://qptm.omicsbio.info).

## 3 Results

### 3.1 A statistical framework for identifying the stable phosphoproteome

**Fig. 1.**
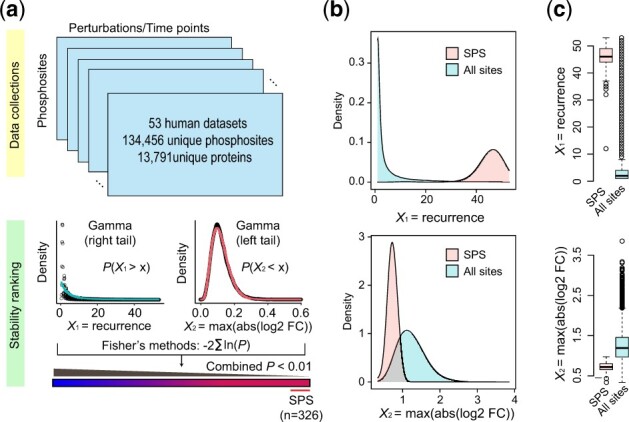
Identification of the stable human phosphoproteome. (**a**) Schematic summary of the framework used for identifying the stable phosphoproteome from across 53 human phosphoproteomic datasets profiling diverse cell types, tissues and perturbations/conditions (see Section 2 for details). (**b**) The distributions of the two stability features, recurrence and phosphorylation changes (maximum of absolute log2 fold change across treatment/conditions), for SPS and all phosphosites. (**c**) Boxplots comparing the two stability features of SPS and all phosphosites, respectively

To generate the stable phosphoproteome, we developed a statistical framework that integrates a large collection of phosphoproteomics datasets and extracts a global profile of SPSs. To ensure that the SPSs we identified accurately represent the stable phosphoproteome, we applied the proposed framework to a comprehensive data collection of 53 human phosphoproteomics datasets covering a total of 40 cell/tissue types and the phosphoproteomic changes across 194 conditions (Yu *et al.*, 2019) (Supplementary Table S1). The final resource contains 134 456 unique phosphosites on 13 791 unique proteins, representing a broad coverage of the human phosphoproteome (Fig. 1a). Specifically, the proposed analytical framework is motivated by the assumption that highly stable sites are those that were frequently identified in phosphoproteomic datasets and with minimal change in phosphorylation levels (Kim *et al.*, 2021), and hence we defined SPSs on the basis of the two criteria: (i) the recurrence of phosphosite identification across phosphoproteomics datasets and (ii) the degree of changes in phosphorylation levels between the basal and perturbations. Since a phosphosite in a given dataset may have multiple treatments/conditions and may be up- or down-regulated, we computed phosphorylation changes as the maximum of the absolute log2 fold-changes across all treatments/conditions. To combine these two stability features, we fitted a gamma distribution to each component for all phosphosites since both features are non-negative with a right-tail distributions. We then applied Fisher’s method to generate a final stability statistic measuring the degree of stability for each of all phosphosites. Using a conservative threshold (*P*-value < 0.01), we obtained a total of 326 phosphosites that were considered as highly SPSs (Fig. 1a) (Supplementary Table S2).

We confirmed that these SPSs have the highest recurrence and the lowest phosphorylation changes among all phosphosites (Fig. 1b and c). Notably, we observed a moderate but statistically significant negative correlation between the recurrence of SPS and the phosphorylation changes (*r* = −0.22; Supplementary Fig. S1a), consistent with our assumption that the two stability features complement each other in defining the stable phosphoproteome. To test the reproducibility of the proposed computational framework, we randomly sub-sampled 80% of the 53 datasets and repeated the computation of the stability statistics multiple times. We observed a strong correlation between the stability statistics from sub-samplings results (*r *=* *0.96; Supplementary Fig. S1b), demonstrating a high reproducibility of the computational framework for stable phosphoproteome identification.

### 3.2 SPSs are stable across various cell types and species

**Fig. 2.**
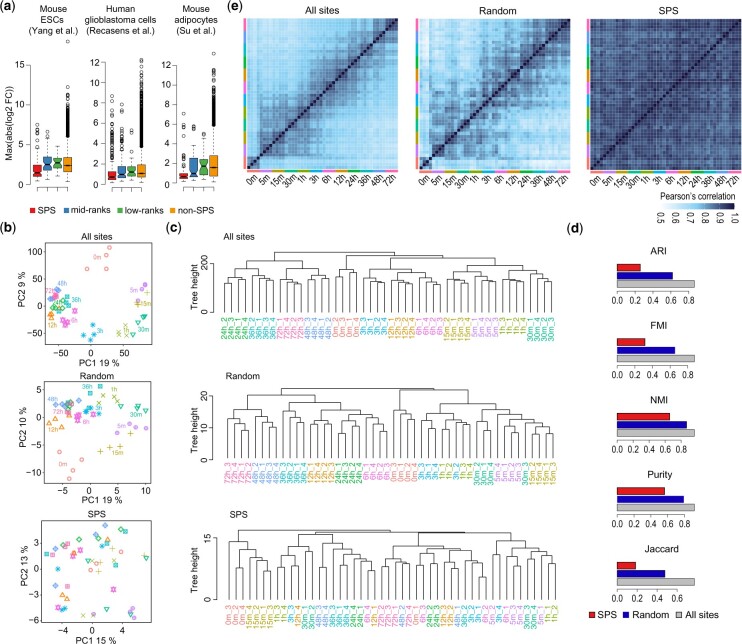
Evaluation of generalizability of stable phosphoproteome. (**a**) Quantification (absolute log2 fold changes) of phosphorylation changes of SPS (red) and phosphosites that are in mid-ranks (blue; same size as SPS), low-ranks (green; same size as SPS) and all phosphosites excluding SPS (light brown) in three ‘independent’ datasets (i.e. human glioblastoma, and mouse ESCs and adipocytes), in that they are not part of the 53 datasets used for deriving SPS. (**b**) PCA visualizing temporal dynamics of global changes in the phosphoproteome of the ESC dataset using all phosphosites, a size-matched random set, or subset by SPSs. (**c**) Hierarchical clustering of biological quadruplicates from the time-course ESC differentiation phosphoproteomic dataset. Top, using all phosphosites in the data; Middle, subsetting the data using the size-matched random set; and bottom, subsetting the data using SPS. (**d**) Numeric quantifications of clustering concordance with sample time point labels from using all sites, the size-matched random set and SPS as in (c) using five concordance measures (i.e. ARI, FMI, NMI, Purity and Jaccard; see Section 2). (**e**) Heatmaps visualizing the correlations among each biological replicates within each time point and across all time points using all phosphosites in the data (left), the size-matched random set (middle) or those subsetted by SPS (right)

We next evaluated our high-confidence SPSs on three independent phosphoproteomics datasets that were not included in the data collection. The datasets consisted of a human glioblastoma inhibition dataset (human glioblastoma) (Recasens *et al.*, 2021) and two murine datasets, an embryonic stem cell differentiation dataset (mouse ESCs) (Yang *et al.*, 2019) and an adipocyte treatment dataset (mouse adipocytes) (Su *et al.*, 2019), which were included to investigate the generalizability of our human SPSs to mouse orthologous phosphosites. We found that SPSs demonstrated the lowest phosphorylation changes in comparison to the size-matched middle- and bottom-ranked phosphosites (i.e. phosphosites ranked in the middle or bottom based on their stability statistics) and non-SPS sites across all the datasets. These findings confirm that SPSs show small phosphorylation changes in phosphorylation upon perturbation across various cell types and suggest that SPSs are conserved across two species, human and mouse (Fig. 2a).

Characteristically, SPSs by nature of their stability are expected to demonstrate a low capacity to discriminate between samples or timepoints. We would therefore expect that if SPSs are used to discriminate phosphoproteomic data covering distinct samples in replicates, they would do poorly to recapitulate the similarity within replicates of the same samples and variability among different samples. To investigate this, we applied PCA and hierarchical clustering on the mESC differentiation dataset using either SPSs, a size-matched random set or all the phosphosites. Using all the phosphosites or the random set, we observed a strong clustering of biological replicates as well as a clear ordering of the time points that were in line with the expected findings. In contrast, neither the clustering of biological replicates nor the trajectory of the differentiation was observed using SPSs (Fig. 2b and c). We observed the same findings in the glioblastoma and adipocyte datasets (Supplementary Figs S2a and b, S3a and b). The results from the hierarchical clustering were quantified in terms of the concordance between the clustering output and the pre-defined labels (conditions or time points) using five performance metrics (ARI, FMI, NMI, purity and Jaccard index; see Section 2). We found that the concordance in clustering, denoted by the evaluation metrics, was much lower when SPSs were used compared with those from using the random set or all phosphosites (Fig. 2d, Supplementary Figs S2c and S3c). Moreover, by visualizing the pairwise correlation of the four biological replicates across all the time points in the mouse ESCs dataset, we observed a stronger correlation of biological replicates and also between closer conditions than distant conditions when using the random set or all the phosphosites. No such pattern was observed using SPSs (Fig. 2e); the lack of contrast in the heatmap further revealed the low variability of SPSs. Lastly, we show that the human orthologous sites of a set of mouse SPSs identified previously (Kim *et al.*, 2021), demonstrated a significantly lower stability index than the rest of the human phosphosites (Supplementary Fig. S4a). Together, these results suggest that SPSs are stably phosphorylated across cell types/tissues and perturbations, and are conserved across species.

### 3.3 SPSs are evolutionarily conserved and functionally important

**Fig. 3.**
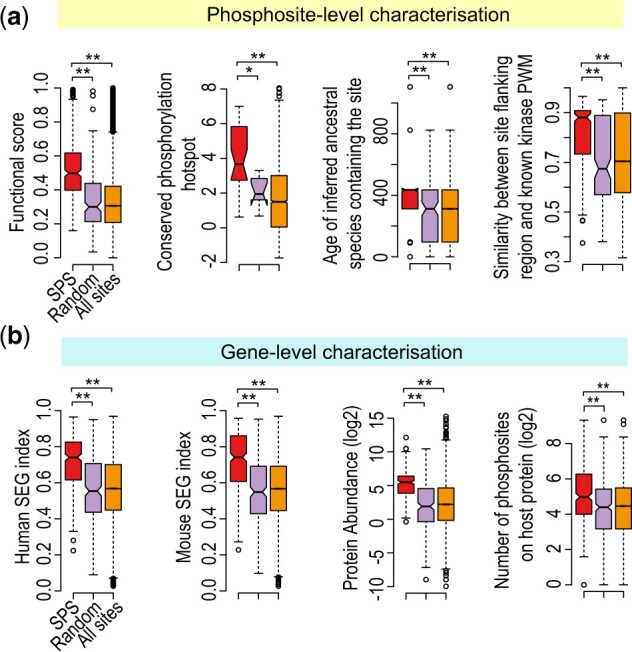
Characterization of SPSs and host genes/proteins for their function and evolution conservation. (**a**) Phosphosite-level characterization using various annotation information derived from Ochoa *et al.* (2020). The ‘Random’ set represents randomly selected phosphosites (from all phosphosites excluding SPS) that match the size of SPS (*n* = 326). *P*-values (*P < 0.05, **P < 0.01) were calculated from a 2-sided Wilcoxon test. (**b**) Gene-level characterization where the host genes/proteins of SPS, random sites as defined in a, and all sites were analyzed for different gene and protein characteristics. *P*-values (*P < 0.05, **P < 0.01) were calculated from a 2-sided Wilcoxon test

To comprehensively characterize SPSs, we assessed its various features on both phosphosite- and protein-level. We first derived the functional scores for each phosphosites from a previous study which examined phosphosites functionality (Ochoa *et al.*, 2020). The functional scores, reflecting the functional importance of phosphorylation sites, were generated by integrating various information covering proteomic, structural, regulatory or evolutionary relevance of phosphosites using a machine learning approach. We found that SPS had significantly higher functional scores relative to either a size-matched random set or the background of all sites (Fig. 3a, first panel). To further investigate the functionality of SPS, on phosphosite level, we examined several features which were highly informative for accurately predicting the functionality of phosphosites. We found that SPS is frequently located at highly conserved phosphorylation hotspots which were identified from 40 eukaryotic species (Strumillo *et al.*, 2019) (Fig. 3a, second panel). Moreover, the older age of their inferred ancestral species (Fig. 3a, third panel) revealed that SPS is evolutionarily conserved.

At the gene level, we found that SPS is preferentially associated with stably expressed genes derived either from mouse or human (Lin *et al.*, 2019) (Fig. 3b, first and second panels), suggesting that SPS host proteins are stably expressed. We also found that the average abundance of SPS host proteins are relatively high (Fig. 3b, third panel). However, phosphosites of highly abundant host proteins are not any more stable compared with those of low abundance (Supplementary Fig. S4b). These data suggest that while SPS host proteins are relatively more abundant, higher protein abundance does not necessarily correlate with higher phosphorylation stability. There were significantly more phosphosites on SPS host proteins than the random set and background (Fig. 3b, fourth panel), suggesting that the host proteins are likely to serve as signaling integrators. Consistent with the site-level analysis, the high conservation score of SPS host genes indicates that they are evolutionarily conserved (Supplementary Fig. S4c, first panel).

**Fig. 4.**
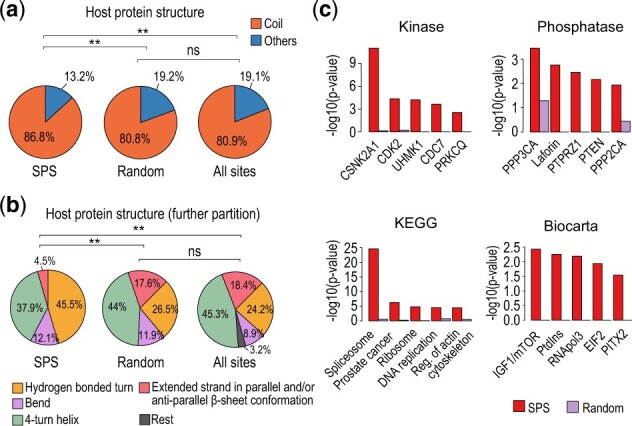
Structural and functional characterization of the SPSs. (**a**) Structural characterization of host proteins as defined in Figure 3b. Protein structure information was derived from Ochoa *et al.* (2020). Differences in proportions were tested by goodness-of-fit using the chi-square test (*P < 0.05, **P < 0.01). (**b**) Distribution of secondary structure of the ‘Others’ category in (a) for SPS, the random set and all sites. (**c**) Overrepresentation analysis (i.e. Fisher’s exact test) of kinases, phosphatases and pathways for SPSs or their host genes

While phosphosites are known to be preferentially located in unstructured coil regions (Iakoucheva *et al.*, 2004; Jiménez *et al.*, 2007), our analysis of the host protein secondary structures showed SPSs have significantly higher preference for unstructured coil regions as compared with the host proteins from the size-matched random set or the background (Fig. 4a). The predictions of structural disorder for phosphosite were consistent with the proportion of secondary structure within host proteins (Supplementary Fig. S4c, second panel): SPSs were preferentially located at disordered regions. This is possibly because phosphosites in unstructured or disorder regions are more accessible to binding by kinases and the recognition of binding motifs in those regions are less dependent on tertiary structure (Landry *et al.*, 2009). For secondary structures associated with ordered and folded regions, SPS was found to be preferentially resided at hydrogen bonded turn and bend rather than helix and beta-sheet (Fig. 4b).

Protein phosphorylation is an important cellular mechanism orchestrated by the activities of kinases and phosphatases (Hunter, 2000) and is important for the regulation of many canonical biological pathways (Proud, 2019). Enrichment of known kinase- and phosphatase-substrate recognition motifs were among the most informative features for accurately predicting the functionality of phosphosites. To this end, we examined the enrichment of SPSs with known kinase- and phosphatase-substrate recognition motifs. The flanking regions of SPSs were found to be significantly better matched to known kinase-substrate recognition motifs (Fig. 3a, fourth panel), and SPSs also had higher probability of being the target sites of kinase-substrate motifs as compared with random sites and the background (Supplementary Fig. S4c, third panel). To further examine the specific kinases and phosphatases that regulate SPS, we performed kinase enrichment on the phosphosite-level (Fig. 4c, top left panel; Supplementary Fig. S5a) and phosphatase enrichment on the protein-level (Fig. 4c, top right panel). We found that SPS were enriched for targets of cell cycle related kinases (CDKs) such as CDK2, CDC7, CDK1, CDK7, CDK6, CDK4, while no significant enrichment was observed from the random set for these kinases (Supplementary Fig. S5a). In addition, SPSs were enriched for targets of several phosphatases, with limited enrichment observed for the random set. Pathway enrichment analysis using KEGG, Biocarta or Reactome annotations showed that SPS host proteins were strongly associated with canonical pathways (e.g. mRNA splicing, mRNA processing), but not for the random set (Fig. 4d, bottom panels; Supplementary Fig. S5b, first panel). Similar enrichments of SPS host proteins among essential cellular activities were observed across the three GO domains (biological process, cellular component and molecular function) (Supplementary Fig. S5b, second to fourth panels). Together, the higher enrichment of kinase- and phosphatase- motifs and canonical pathways among SPSs indicates that they are likely to play central roles in the regulation of cellular functions.

### 3.4 SPSs are enriched in spliceosomes and frequently affected in cancer

**Fig. 5.**
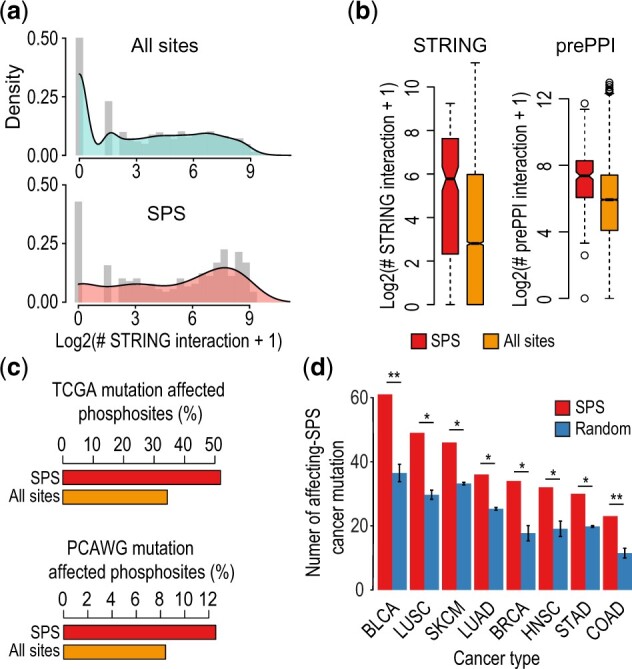
SPS enrichment in PPIs and association with cancer mutation. (**a**) Distributions of number of PPIs (derived from STRING database) for host proteins of SPSs (red) and all phosphosites (light blue). (**b**) The number of PPIs (log2 transformed) for the host proteins of SPSs and all sites identified from either STRING or prePPI databases. (**c**) The percentages of cancer mutation-affected SPSs in comparison to all phosphosites using either TCGA or PCAWG databases. (**d**) The mutation frequency of SPS and size-matched random sets by cancer types (*P < 0.05, **P < 0.01)

Dysregulation of phosphorylation has been linked to several human diseases, including numerous cancers (Rikova *et al.*, 2007; Sever and Brugge, 2015). Mechanistically, mutations nearby phosphorylation sites can affect the physicochemical properties of the flanking regions around the residue, thus change the interactions of the host proteins with other proteins, the binding preference with kinases, or abolish kinase binding, and therefore may rewire signaling networks involved in cancer progression (Lundby *et al.*, 2019). We first investigated whether SPSs are signaling hubs within the PPI networks and found that compared with the background, SPSs have significantly more PPIs derived from either STRING (Szklarczyk *et al.*, 2019) or prePPI database (Zhang *et al.*, 2013) (Fig. 5a and b), indicating SPS hosts are hub proteins with high number of interaction partners. We next performed the enrichment analysis of the CGNs as defined in the Molecular Signatures Database (Liberzon *et al.*, 2015). We found that SPS host genes are significantly enriched for cancer-associated genes compared with the host genes of the size-matched random phosphosites (Supplementary Fig. S5c). We then analyzed the SPSs for their susceptibility to cancer mutations. We found that a large proportion of SPSs are annotated as affected by cancer mutations compared with the background using either TCGA or PCAWG databases (Krassowski *et al.*, 2021) (Fig. 5c). Finally, we further categorized the mutation frequency of SPS and the size-matched random sets by eight different cancer types. We found that cancer mutation affected SPS were more frequently detected across different cancer types compared with those of random sets (Fig. 5d). Together, these results suggest that many host genes of SPSs are the key nodes in PPI networks and are associated with CGN, and the SPSs themselves are frequently affected by cancer mutations across cancer types.

**Fig. 6.**
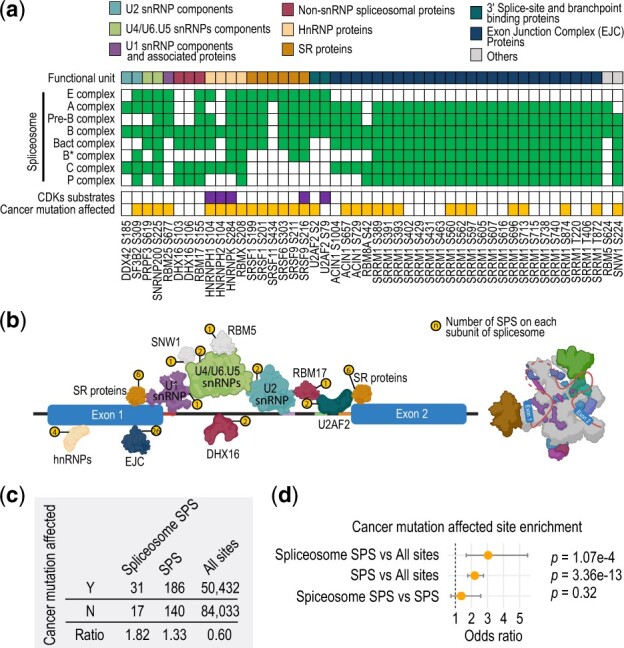
Association of spliceosome SPSs with cancer mutation. (**a**) Annotation of spliceosome SPSs. Sites are grouped into functional units and green boxes in the heatmap denote the inclusion of SPS host proteins in each of the spliceosome complexes. Sites that are known to be the substrates of CDKs are highlighted in purple and those that are affected by cancer mutations are highlighted in yellow. (**b**) Schematic summary illustrating the number of SPSs on each subunit of spliceosome. (**c, d**) Ratios of cancer mutation affected sites in spliceosome SPS, SPS and all sites (c) and odds ratio tests comparing the three sets (d). Error bars represent 95% confidence intervals

Given that SPS host genes are enriched for splicing-related pathways and, in particular, spliceosome (Fig. 4c and Supplementary Fig. S5b) and the changes with alternative splicing are frequently linked to cancer (David and Manley, 2010; Zhang *et al.*, 2021), we sought to investigate whether SPSs are associated with cancer by rewiring the signaling of splicing factors in the spliceosome. To this end, we focused on SPSs whose host proteins are associated with the spliceosome complex (Will and Lührmann, 2011). Out of 326 SPSs, 48 of them (15%) are within 21 spliceosome-associated proteins (refer to as spliceosome SPSs hereafter), covering eight functional categories (Fig. 6a and Supplementary Table S2). Furthermore, the vast majority of these spliceosome proteins (19 out of 21) are associated with at least three different spliceosomal complexes, suggesting the potential impact of the spliceosome SPSs may have on multiple stages of splicing. Notably, most spliceosome SPSs are concentrated on a few subunits including SR proteins, exon junction complex (EJC) and heterogeneous nuclear ribonucleoproteins (hnRNPs) (Fig. 6b). Specifically, the SR protein SRSF1 is known to mediate spliceosome assembly and is essential for nuclear import (Zhong *et al.*, 2009). In addition, hyperphosphorylation of SRSF1 at multiple sites including SPSs at serine 199 and 201, via oncogenic activation of PI3k/Akt pathway, could result in differential alternative splicing of Casp9 which favors the prosurvival Casp9b isoform over the proapoptotic Casp9a isoform in non-small cell lung cancers (Shultz *et al.*, 2010). The EJC serves as an anchor in splicing for various processing proteins (Le Hir *et al.*, 2001) and the peripheral EJC component SRRM1, containing more than 20 SPSs, has been demonstrated that its phosphorylation status influences splice-site selection (Cheng and Sharp, 2006) and SRRM1 overexpression has been associated with the aggressiveness of prostate cancer (Jiménez-Vacas *et al.*, 2020). Finally, hnRNPs are well-characterized as splicing silencers (Wang *et al.*, 2011). Phosphorylations of HNRNPK at serines 284, a SPS, among other sites were reported to regulate its nucleocytoplasmic localization and activity (Habelhah *et al.*, 2001), and the dysregulation of HNRNPK is a hallmark of poor prognosis in multiple cancers (Carpenter *et al.*, 2006).

Given the potential impact SPSs may have on spliceosome assembly, localization and RNA splicing, and that a large proportion of spliceosome SPSs are affected by cancer mutations (Fig. 6a), we next compared the proportion of cancer mutation-affected spliceosome SPSs, all SPSs and all phosphosites. We found that while cancer mutation-affected phosphosites were significantly enriched in SPSs (odds ratio = 2.2), the enrichment in spliceosome SPS is even more substantial (odds ratio = 3.0) (Fig. 6c and d). Taken together, these results highlight the possible impact of SPSs on splicing factors in regulating RNA splicing and point to a potential link between their dysregulation, such as those due to mutations, in the spliceosome complex and the onset of oncogenic processes.

## 4 Discussion and conclusion

Our search for SPSs was initially guided by their utility in phosphoproteomic data normalization and batch correction (Kim *et al.*, 2021). Nevertheless, the conceptual similarity between SPSs and stably expressed housekeeping genes, which are indispensable in a wide range of cell/tissue types (Lin *et al.*, 2019), led us to wonder about their biological importance and the essential roles they may play in cell signaling and disease. Motivated by this quest, we developed a statistical framework to systematically identify the stable phosphoproteome from a large collection of human phosphoproteomic datasets that profiled a diverse set of cell/tissue types. Our statistical framework identified a total of 231 SPS host proteins each containing on average 32 phosphosites and out of which 1.4 are SPSs, suggesting that only a small percentage (4%) of the phosphosites on SPS host proteins (i.e. genes whose protein-product contains one or more SPSs) are stable. While we found that SPS host genes tend to be stable in their expression (Fig. 3b), these statistics suggest that the stability of SPSs are unlikely to be explained solely by the stability of their host genes/proteins but should be attributed to their stability in phosphorylation regulation.

To validate the reproducibility of SPSs identified from the 53 phosphoproteomics datasets, we have performed a subsampling analysis (Supplementary Fig. S1b) and have also repeated the analysis using an independent set of datasets (Supplementary Fig. S1c). While these results suggest that SPSs can be identified with high reproducibility, we acknowledge that the 53 datasets used in this work cannot represent the full variety of conditions and cell/tissue types and hence will not fully determine the landscape of the stable phosphoproteome. Future work is required to further explore how characteristics such sex, cell type, immortalization status and diseases may affect the generalization of results from current datasets. The landscape of the stable phosphoproteome. Future work is required to further explore how characteristics such sex, cell type, immortalization status and diseases may affect the definition of SPSs and the generalization of results from current datasets.

Most phosphoproteomics studies have so far focused on identifying dynamically regulated phosphosites between cell types and conditions while ignoring phosphosites that are stable presumably under the assumption that they lack functions. Our analysis, however, sheds light on a highly stable phosphoproteome that is evolutionarily conserved and functionally important. One explanation of the high stability found in these phosphosites across various cellular systems is that they are so essential that dysphosphorylation of the SPS would lead to significant disruption on the core cellular processes, resulting in the diseases such as cancer. Indeed, our characterization of SPSs highlights their enrichment in proteins/pathways associated with RNA splicing, an essential cellular process in mammalian cells, and suggests a potential link between the dysregulation of spliceosomes and cancer via mutations on spliceosome SPSs. While increasing evidence demonstrates that mis-splicing contributes to cancer progression (Du *et al.*, 2021; Scotti and Swanson, 2016), the functional significance of PTM on splicing factors and their relationship with cancers remains uncharacterized. Our analysis links stable phosphorylation sites on splicing factors to cancer mutations allowing us to contemplate a common mechanism in cancer development through targeting the core phosphoproteome of mammalian cells.

## Funding

This work was supported by a National Health and Medical Research Council (NHMRC) Investigator [1173469 to P.Y.], Children’s Medical Research Institute Postgraduate Scholarships to D.X. and H.J.K., and an Australian Research Council (ARC) Postgraduate Research Scholarship to H.J.K. and Luminesce Alliance—Innovation for Children’s Health established with the support by the NSW Government. 

*Conflict of Interest*: none declared. 

## Supplementary Material

btac015_Supplementary_Data
