# Supplementary material for: Functional analysis of the stable phosphoproteome reveals cancer vulnerabilities
Source: Bioinformatics. 2022 Jan 7;38(7):1956–63. doi: 10.1093/bioinformatics/btac015 (PMC9113330; doi:10.1093/bioinformatics/btac015)
Supplement: btac015_Supplementary_Data [file btac015_supplementary_data.zip › btac015-suppl_data/Supplemental figures_Bioinformatics.docx]

**Supplementary figures**

**Supplementary Figure 1. Quality and reproducibility controls of stable phosphoproteome.** (**a**) Scatter plot of recurrence and phosphorylation changes across all phosphosites. Pearson’s correlation was used to quantify the correlation between the two stability features. (**b**) Scatter plot of stability statistics derived from two representative runs of the proposed computational framework using random sub-samples of 42 datasets (80% from all 53 datasets). Pearson’s correlation was used to quantify the correlation between the two sets of stability statistics. (**c**) Scatter plot of stability statistics derived from proposed computational framework using original 53 datasets or additional 26 independent datasets (see Methods). Pearson’s correlation was used to quantify the correlation between the two sets of stability statistics.


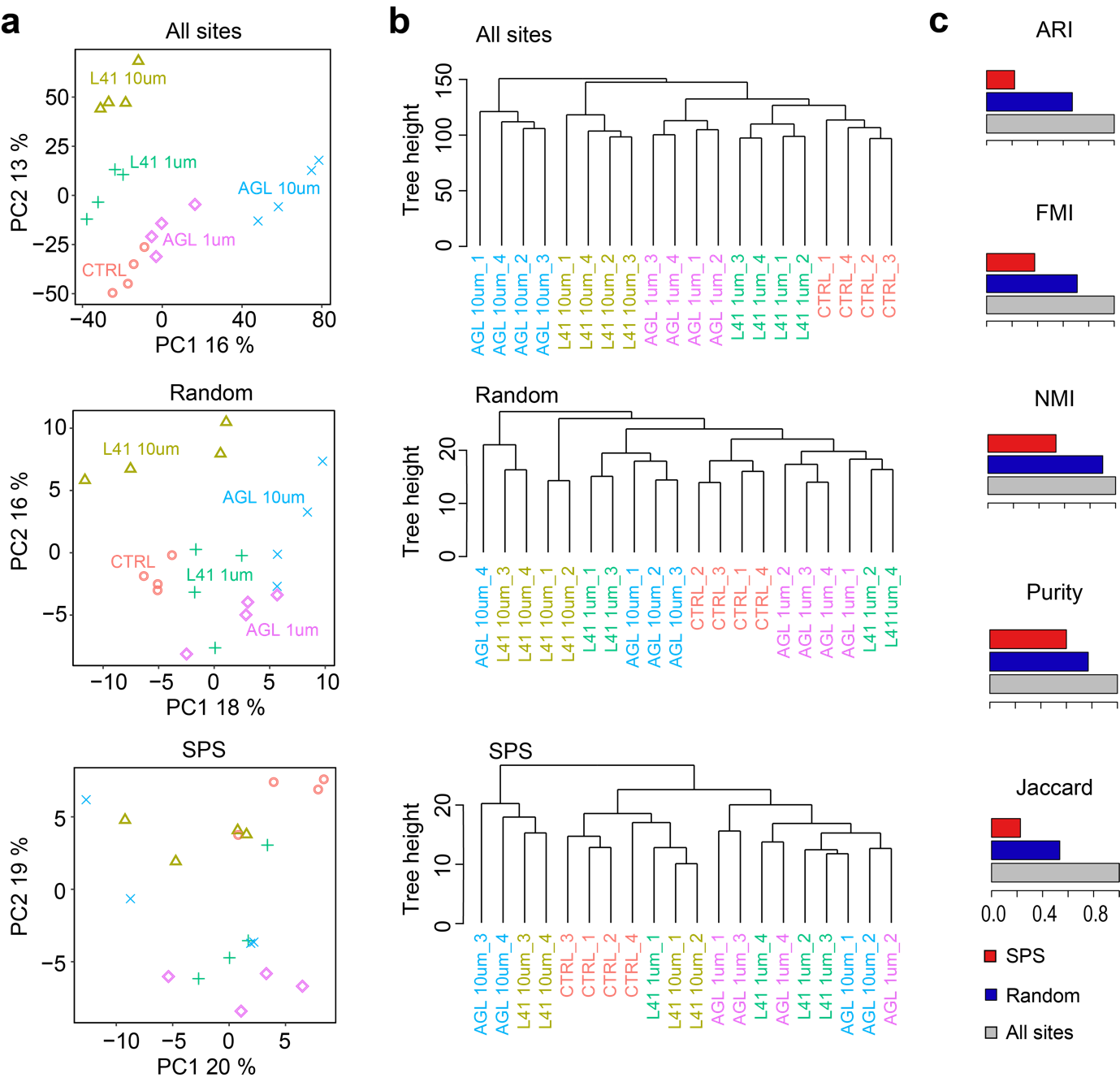


**Supplementary Figure 2. Evaluation of SPS on human glioblastoma dataset. (a)** Principal component analysis (PCA) visualising phospho-dynamics of the human glioblastoma dataset using either all phosphosites, subset by the size-matched random set, or subset by SPSs. Each circle represents a sample and the colours denote treatments and the control. **(b)** Hierarchical clustering of biological quadruplicates from the glioblastoma phosphoproteomic dataset. Top, using all phosphosites in the data; middle, subsetting the data using the size-matched random set; bottome, subsetting the data using SPSs. **(c)** Numeric quantifications of clustering concordance with sample condition labels from using all sites, the size-matched random set and SPSs as in **(b)** using five concordance measures (i.e., ARI, FMI, NMI, Purity, and Jaccard; see Methods).


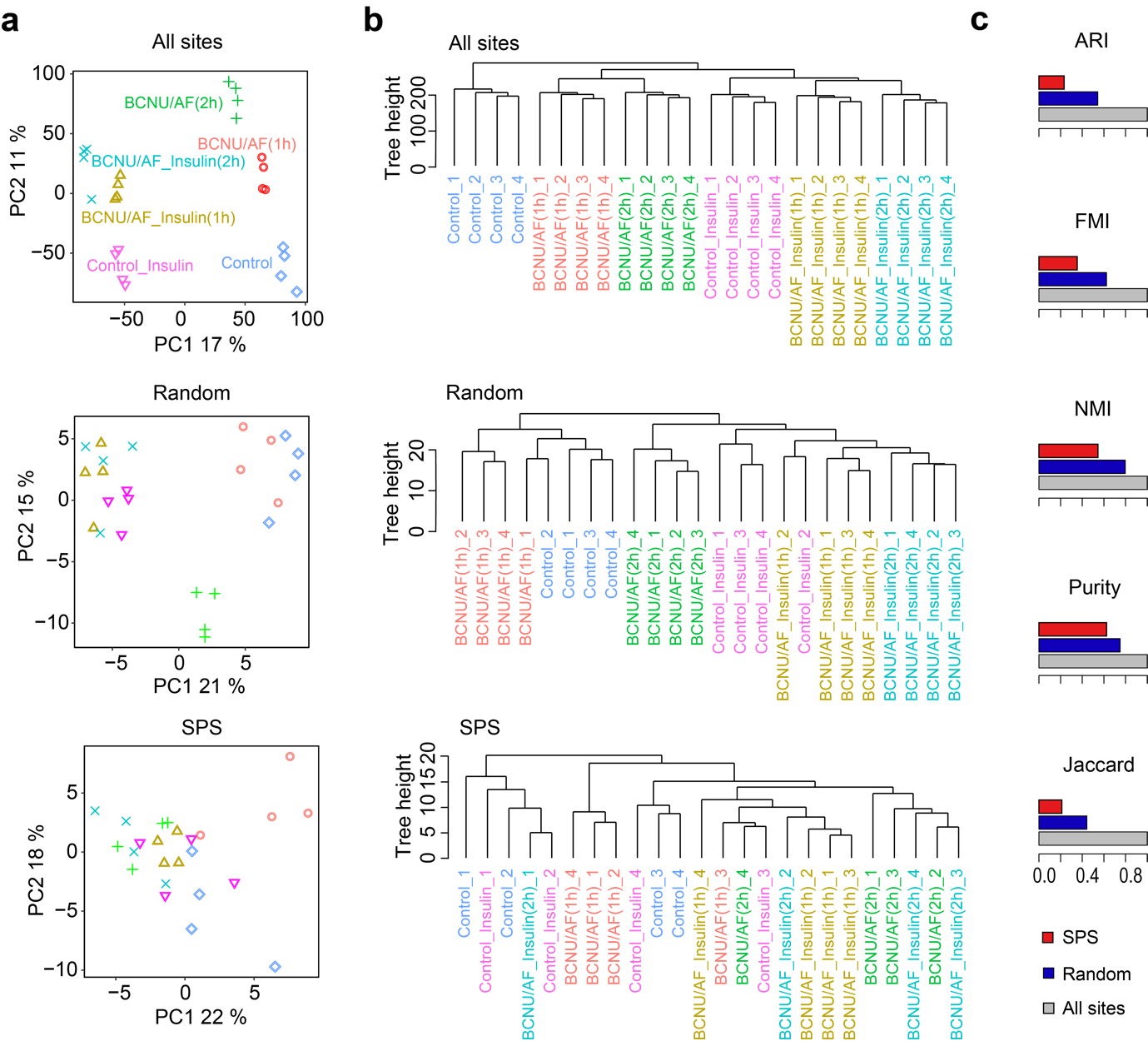


**Supplementary Figure 3. Evaluation of SPS on mouse adipocyte stimulation dataset. (a)** Principal component analysis (PCA) visualising phospho-dynamics of the mouse adipocyte dataset using either all phosphosites, subset by the size-matched random set, or subset by SPSs.. Each circle represents a sample and the colours denote treatments and controls. **(b)** Hierarchical clustering of biological quadruplicates from the adipocyte phosphoproteomic dataset. Top, using all phosphosites in the data; middle, subsetting the data using the sized-match random set and bottom, subsetting the data using SPSs. **(c)** Numeric quantifications of clustering concordance with sample condition labels from using all sites, the sized-match random set and SPS as in **(b)** using five concordance measures (i.e., ARI, FMI, NMI, Purity, and Jaccard; see Methods).

**Supplementary Figure 4. Characterisation of SPSs for their function and evolution conservation. (a)** Boxplots of human phosphosite stability indexes for mouse SPSs (orthologous), derived from [(Kim et al. 2021)](https://paperpile.com/c/54MANM/5OqS), and all sites. **(b)** Stability index of top-50 phosphosites with most abundant host proteins and bottom-50 phosphosites with least abundant host proteins. **(c)** Phosphosite- or gene-level characterisation using annotation information derived from [(Ochoa et al. 2020)](https://paperpile.com/c/2lqpl9/4VX6). The ‘Random’ set represents randomly selected phosphosites (from all phosphosites excluding SPS) that match the size of SPSs (n=326). All statistical tests were performed using Wilcoxon rank-sum test (** p<0.01; ‘ns’ denotes p>0.05).


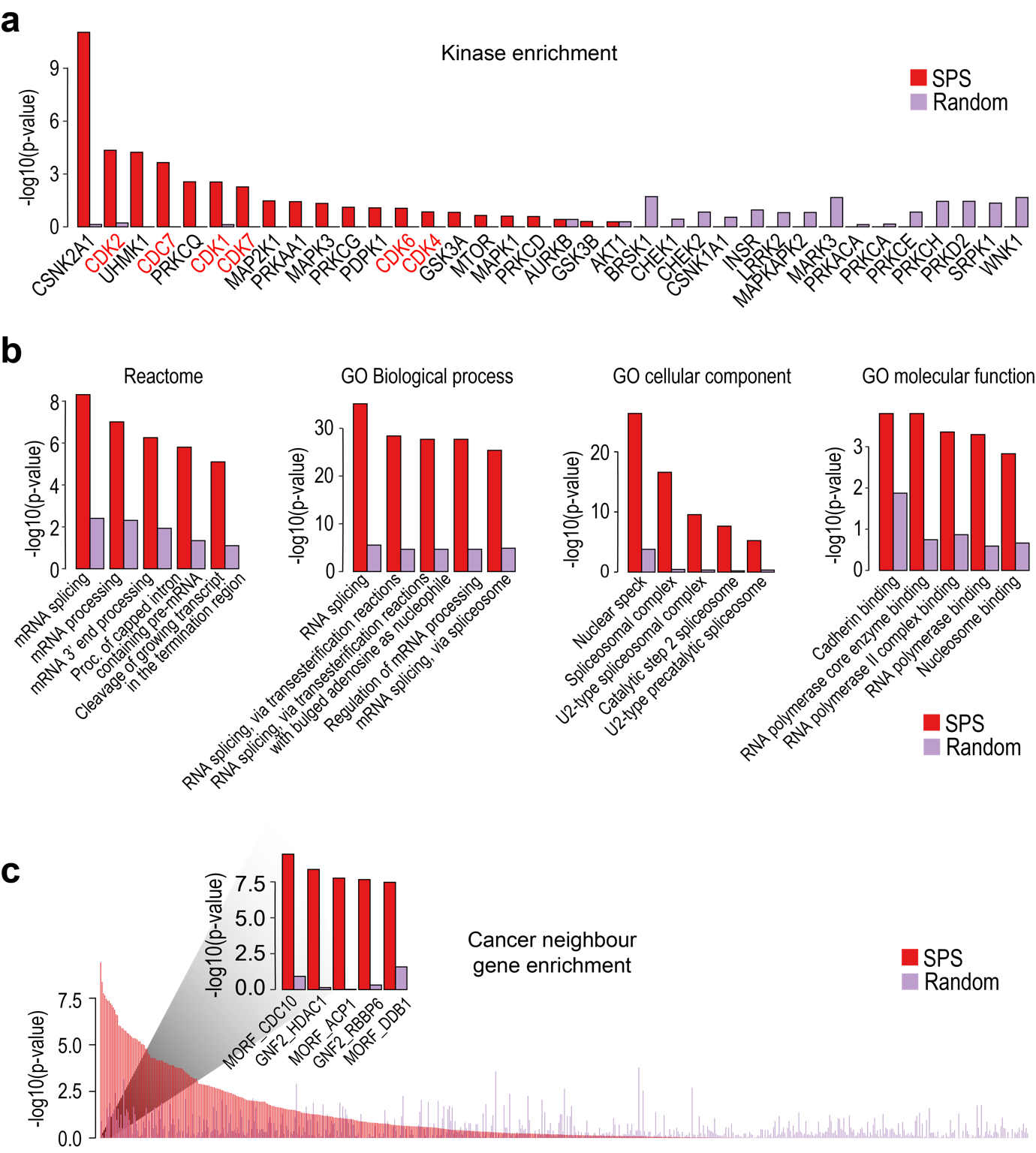


**Supplementary Figure 5. Assessment of SPSs for their function, interaction, and cancer association.** **(a)** Overrepresentation analyses of kinases for the host genes of SPSs and the size-matched random sites. **(b)** Overrepresentation analyses of pathways annotated in the Reactome and the GO databases (including biological process, cellular component and molecular function) for the host genes of SPSs and the size-matched random sites. **(c)** Overrepresentation analyses of cancer gene neighbours for the host genes of SPSs and the size-matched random sites (see Methods for details).
